# Trends in smoking initiation in Europe over 40 years: A retrospective cohort study

**DOI:** 10.1371/journal.pone.0201881

**Published:** 2018-08-22

**Authors:** Alessandro Marcon, Giancarlo Pesce, Lucia Calciano, Valeria Bellisario, Shyamali C. Dharmage, Judith Garcia-Aymerich, Thorarinn Gislasson, Joachim Heinrich, Mathias Holm, Christer Janson, Deborah Jarvis, Bénédicte Leynaert, Melanie C. Matheson, Pietro Pirina, Cecilie Svanes, Simona Villani, Torsten Zuberbier, Cosetta Minelli, Simone Accordini, on behalf of the Ageing Lungs In European Cohorts study

**Affiliations:** 1 Unit of Epidemiology and Medical Statistics, Department of Diagnostics and Public Health, University of Verona, Verona, Italy; 2 Department of Public Health and Pediatrics, University of Turin, Turin, Italy; 3 Allergy and Lung Health Unit, School of Population and Global Health, The University of Melbourne, Melbourne, Australia; 4 ISGlobal, Centre for Research in Environmental Epidemiology (CREAL), Barcelona, Spain; 5 Universitat Pompeu Fabra (UPF), Barcelona, Spain; 6 CIBER Epidemiología y Salud Pública (CIBERESP), Barcelona, Spain; 7 Department of Respiratory Medicine and Sleep, Landspitali University Hospital (E7), Reykjavik, Iceland; 8 University of Iceland, Faculty of Medicine, Reykjavik, Iceland; 9 Institute and Outpatient Clinic for Occupational, Social and Environmental Medicine, Clinical Center, Ludwig Maximilians University, Comprehensive Pneumology Centre Munich, German Centre for Lung Research, Muenchen, Germany; 10 Occupational and Environmental Medicine, Sahlgrenska Academy, University of Gothenburg, Gothenburg, Sweden; 11 Department of Medical Sciences: Respiratory, Allergy and Sleep research, Uppsala University, Uppsala, Sweden; 12 Population Health & Occupational Disease, National Heart and Lung Institute, Imperial College London, London, United Kingdom; 13 MRC-PHE Centre for Environment and Health, Imperial College London, London, United Kingdom; 14 Inserm UMR 1152, Pathophysiology and Epidemiology of Respiratory Diseases, Paris, France; 15 University Paris Diderot Paris 7, UMR 1152, Paris, France; 16 Institute of Respiratory Diseases, University of Sassari, Sassari, Italy; 17 Centre for International Health, University of Bergen, Bergen, Norway; 18 Department of Occupational Medicine, Haukeland University Hospital, Bergen, Norway; 19 Unit of Biostatistics and Clinical Epidemiology, Department of Public Health, Experimental and Forensic Medicine, University of Pavia, Pavia, Italy; 20 Allergy Centre Charité, Department of Dermatology & Allergy, Charité—Universitätsmedizin Berlin, Berlin, Germany; Legacy, Schroeder Institute for Tobacco Research and Policy Studies, UNITED STATES

## Abstract

**Background:**

Tobacco consumption is the largest avoidable health risk. Understanding changes of smoking over time and across populations is crucial to implementing health policies. We evaluated trends in smoking initiation between 1970 and 2009 in random samples of European populations.

**Methods:**

We pooled data from six multicentre studies involved in the Ageing Lungs in European Cohorts consortium, including overall 119,104 subjects from 17 countries (range of median ages across studies: 33–52 years). We estimated retrospectively trends in the rates of smoking initiation (uptake of regular smoking) by age group, and tested birth cohort effects using Age-Period-Cohort (APC) modelling. We stratified all analyses by sex and region (North, East, South, West Europe).

**Results:**

Smoking initiation during late adolescence (16–20 years) declined for both sexes and in all regions (except for South Europe, where decline levelled off after 1990). By the late 2000s, rates of initiation during late adolescence were still high (40–80 per 1000/year) in East, South, and West Europe compared to North Europe (20 per 1000/year). Smoking initiation rates during early adolescence (11–15 years) showed a marked increase after 1990 in all regions (except for North European males) but especially in West Europe, where they reached 40 per 1000/year around 2005. APC models supported birth cohort effects in the youngest cohorts.

**Conclusion:**

Smoking initiation is still unacceptably high among European adolescents, and increasing rates among those aged 15 or less deserve attention. Reducing initiation in adolescents is fundamental, since youngsters are particularly vulnerable to nicotine addiction and tobacco adverse effects.

## Introduction

Smoking is still the leading cause of avoidable mortality and the strongest modifiable risk factor for respiratory and allergic diseases, cardiovascular diseases, and cancer.[1–3] The prevalence of smoking has been, and still is, declining in Europe, which is mainly related to more restrictive national regulations that have been introduced over time.[1,4,5] The Framework Convention on Tobacco Control is giving further boost to the global fight against smoking.[2] Still, more needs to be done, and understanding trends of tobacco habits over time and across populations is essential to implementing further effective public health policies.

The prevalence of smoking within a population depends on the rates of smoking initiation, cessation, and smoking-related mortality. Most scientific publications on tobacco use report prevalence figures, and only few provide data on age at smoking initiation.[1,2,4,6–8] Teasing out the trends in smoking initiation is especially important to develop primary preventive strategies.

According to the 2015 Eurobarometer special report, 19% of European smokers start before the age of 15 years.[1] Early tobacco exposure is particularly damaging, as individuals are more vulnerable to tobacco effects in the stages of growth than when organ systems are fully developed, and emerging evidence suggests that exposure in early puberty may have an impact on health across generations.[9–11] Young adolescents are particularly susceptible to nicotine addiction.[12] The World Health Organization recommends monitoring tobacco use in the 13–15 year-old population, but data on smoking in early adolescence are still scarce.[1,2,6,7,13] In 2013, the World Health Assembly endorsed an action for a 30% worldwide reduction in the prevalence of smoking by 2025, but young adolescents were not mentioned, which may be related to the lack of data in this age group.[14]

Given the gap in knowledge on this important public health issue, we investigated the time trends in the uptake of regular smoking over 1970–2009 in Europe. We aimed at identifying differences in time trends across age groups by using both age-stratified analysis and Age-Period-Cohort (APC) modelling.

## Methods

### Study design and population

We used the data from six large-scale multicentre studies on random samples of the general population in Europe, which were available in the Ageing Lungs in European Cohorts (ALEC) consortium (http://www.alecstudy.org/) (S1 Fig).

The European Community Respiratory Health Survey (ECRHS) is an international cohort study performed on subjects aged 20–44 years at enrolment in 1991–1994.[15] In ECRHS I, random samples of subjects participating in a postal screening (stage 1) took part in a clinical interview (stage 2), where they reported information on their smoking habits for the first time. The cohort of participants in stage 2 (labelled “ECRHS clinical” throughout the manuscript) was reassessed at two independent follow-up examinations during 1999–2002 in ECRHS II,[16] and during 2010–2013 in ECRHS III (www.ecrhs.org). ECRHS-Italy is the postal follow-up of the Italian participants in ECRHS I stage 1 carried out in 1998–2001 and 2008–2009.[17] The Respiratory Health in Northern Europe (RHINE) study is the postal follow-up of the ECRHS I stage 1 participants from the Nordic centres.[18] We used the RHINE data collected in 2010–2012 only, because age at initiation had been assessed using a different question in 1999–2001. S2 Fig describes how ECRHS clinical, ECRHS Italy and RHINE stem from the ECRHS study. The Global Allergy and Asthma Network of Excellence (GA^2^LEN) study is an international cross-sectional study carried out in Europe in 2007–2009.[19] The Italian Study on Asthma in Young Adults (ISAYA) is a cohort study (baseline: 1998–2000; follow-up: 2008–2009), and the Gene Environment Interactions in Respiratory Diseases study (GEIRD) is a cross-sectional study carried out in Italy (2005–2010).[17,20]

We used data on smoking from postal questionnaires for all studies, in addition to data from clinical interviews in ECRHS. Subjects who had participated in more than one study (or more than one occasion in multi-wave studies) were only considered once.

In each study, one or more random samples of the population were available per centre (S1 Table). In the main analysis, we included subjects from 79 samples from 17 countries with participation rates above 25% (Table 1), and discarded four samples with lower participation rates. We classified the samples into four regions corresponding to the United Nations geoscheme and tobacco epidemiology:[21] North Europe (Denmark, Finland, Iceland, Norway, Sweden, UK), East Europe (Estonia, Macedonia, Poland), South Europe (Italy, Portugal, Spain) and West Europe (Belgium, France, Germany, Netherlands, Switzerland).

**Table 1:** Number of samples, participation rates, and characteristics of participants by region.

European region	North	East	South	West	Overall
Samples^a^ (n)	24	6	35	14	79
Participation rate^b^, % (median, min–max)	53.0 (27.2–83.1)	42.4 (25.4–80.4)	61.9 (32.7–82.2)	50.8 (27.9–67.1)	54.4 (25.4–83.1)
Subjects (n)	50,576	10,556	41,794	16,178	119,104
Males (%)	45.9	44.9	48.2	45.8	46.6
Birth cohort, year (median, min–max)	1962(1908–1997)	1964(1932–1993)	1966 (1923–1997)	1961 (1920–1992)	1964 (1908–1997)
Age^c^, year (median, min–max)	44(11–100)	44 (14–76)	36 (11–86)	37 (15–88)	39 (11–100)
Study (%)					
ECRHS clinical	9.8	3.9	6.8	34.9	11.6
ECRHS Italy	-	-	8.8	-	3.1
RHINE	18.0	8.6	-	-	8.4
GA^2^LEN	72.2	87.5	7.6	65.1	50.0
ISAYA	-	-	44.8	-	15.7
GEIRD	-	-	32.0	-	11.2
Ever smokers (%)	43.7	50.8	48.1	54.7	47.4
Age at starting smoking, year (mean±SD)	17.0±4.2	18.7±4.4	17.3±3.6	17.2±4.2	17.2±4.1
Total years at risk (in age range 11–35 years)	754,567	152,174	621,067	215,419	1,743,227

^a^ original study samples, which correspond to centres (or centres crossed by age group in GEIRD); see S1 Table

^b^ participation rates at the first wave (baseline) for studies with follow up data. In the case of ECRHS, ECRHS-Italy and RHINE, which were follow-up studies of ECRHS I stage 1 (S2 Fig), participation rates were obtained by multiplication assuming independence of participation between ECRHS I stage 1 and the consecutive study

^c^ age at baseline for studies with follow up data

### Data on smoking

We defined “ever smoking regularly” using the question “Have you ever smoked for as long as a year?”, except for RHINE where subjects identified themselves as “smokers” or “ex-smokers” without reference to duration (“for as long as a year”) (Table 2). Each study reported slightly different instructions, however most questionnaires specified that “ever smoking for as long as a year” referred to having smoked “at least one cigarette per day or one cigar per week for one year”. In all the studies, age at initiation was collected only among ever smokers using the question “How old were you when you started smoking?”; we used the first information available for subjects with follow-up data.

**Table 2:** Questionnaire items on smoking.

Study	Smoking status	Age at initiation	Smoking intensity
**ECRHS I, II, III** (clinical interviews)	**Have you ever smoked for as long as a year?** ('YES' means at least 20 packs of cigarettes or 12 oz (360 grams) of tobacco in a lifetime, or at least one cigarette per day or one cigar per week for one year)	**How old were you when you started smoking?**	**How much do you now smoke on average?** (number of cigarettes per day). **On average of the entire time you smoked, before you stopped or cut down, how much did you smoke?** (number of cigarettes per day)
**ECRHS III** (postal questionnaire)	**Have you ever smoked for as long as a year?**		N.A.
**ECRHS Italy, ISAYA, GEIRD**	**Have you ever smoked for as long as a year?** ('YES' means at least one cigarette per day or one cigar per week for one year)		**On average how much do you (or did you) smoke?** (cigarettes per day)
**RHINE**	**Are you a smoker?** (this applies even if you only smoke the odd cigarette/cigar or pipe every week). **Are you an ex-smoker?**		**Smoke/smoked** (cigarettes/week)
**GA**^**2**^**LEN**	**Have you ever smoked for as long as a year?** ('YES' means at least one cigarette per day or one cigar per week for one year)		**On average how much do you (or did you) smoke?** (cigarettes per day)

In order to assess the impact of the retrospective assessment of smoking initiation, we compared age at initiation at different waves of cohort studies (S1 Appendix). This analysis showed a fairly good correlation of age at initiation reported up to twenty years apart, and that precision was higher when age at initiation was derived using the first information available.

### Statistical analysis

We performed both the age-stratified analysis and APC modelling on data pooled across all the studies, with the analyses stratified by sex and European region. Subjects with missing information on smoking status or age at initiation were deleted listwise. All the analyses were performed using STATA 14 (Stata Corp. College Station, TX, USA).

Rates of smoking initiation (per 1000/year) were calculated retrospectively from childhood to the most recent assessment, as the ratio between the number of incident smokers (subjects taking up regular smoking) and total time at risk (person-years). Subjects were considered at risk from age 11 years to age at initiation, age at the last study, or age 35, whichever came first, since our data showed that 0.9% of smokers started before the age of 11, and only 0.7% after the age of 35.

#### Age-stratified analysis

We estimated smoothed trends in smoking initiation rates (with 95% confidence intervals) over the period 1970–2009, using two-level mixed-effects generalized linear models with subjects nested into samples. We used a negative binomial outcome distribution, a logarithmic link function, and an offset for log person-years. These analyses were further stratified into three age groups, which we refer to as young adolescents (11–15 years), late adolescents (16–20 years), and young adults (21–35 years), resulting in 24 separate models (2 genders × 4 regions × 3 age groups). Period (time) was modelled using natural splines with four equally spaced inner knots (i.e. placed to ensure an equal number of new smokers in each sub-period), since four knots provided the best fitting according to the Bayesian Information Criterion (BIC) across most models. Age was included to adjust for potential residual confounding within age groups.

We carried out sensitivity analyses (data not shown) where we (i) excluded the data from RHINE in order to assess the impact of the different question used to define ever smoking, (ii) excluded 14 samples with a response rate below 40%, and (iii) only considered smokers who had reported >5 cigarettes/day (n = 46,382, 82.2% of all smokers) for the calculation of the rates. This was done through right-censoring of subjects with a smaller (n = 6,123) or missing (n = 3,897) number of cigarettes/day at all the available study waves, because for these subjects we might have missed a period of greater smoking intensity between initiation and the time when they answered the questionnaires. These subjects contributed to person-years up to the age at censoring, but they did not contribute to the number of events.

#### APC modelling

We estimated birth cohort effects using APC modelling, assuming that cohort effects resulted from the interaction between age effects and period effects.[22] Under this assumption, a cohort effect arises when a change in some determinants of the variable of interest over time (period effect) affects age groups differently. For example, the enforcement of laws restricting tobacco sales to minors may reduce smoking initiation in children but not in adults. APC modelling was used to test whether differences in trends at different ages (cohort-effects) were statistically significant with no *a priori* assumptions on what age groups are relevant for comparison.

Due to an insufficient number of smokers for some age-period groups in some samples, we did not use multi-level models but merged data from different samples and used single-level regression analysis. APC modelling was carried out in two steps: (i) we first fitted negative binomial regression models on age and period, using natural splines with four and five knots respectively, and calculated model deviance residuals (multi-level models did not provide normally-distributed residuals); (ii) we then analysed the residuals using linear regression on birth cohort (natural spline with three knots), with robust standard errors to account for deviations from normality, and weights inversely proportional to the variance, since the precision of residuals varied according to the number of subjects by age and period. The number of knots for splines was selected based on the BIC as mentioned before.

### Ethics approval and consent to participate

Ethical approval was not requested for this secondary analysis of pooled data from previous studies. In each of the original studies, ethical approval was obtained for each centre from the appropriate ethics committee.[15–20] All procedures have conformed to the principles embodied in the Declaration of Helsinki. Written informed consent was obtained from participants in the clinical examinations (ECRHS clinical). In ECRHS Italy, RHINE, GA^2^LEN, ISAYA and GEIRD, only data from postal questionnaires were used, which were voluntarily sent back giving consent to use the anonymized data.

## Results

The combined dataset included 55,490 males (780,068 person-years) and 63,614 females (975,609 person-years). GA^2^LEN was the largest study, while ECRHS contributed with the largest number of samples (Table 1). The median age of the participants ranged from 33 years in ECRHS and ISAYA, to 52 years in RHINE. The prevalence of ever smoking ranged from 42.8% in GA^2^LEN to 61.3% in ECRHS (S2 Table).

### Time trends in smoking initiation (age-stratified analysis)

**Fig 1:**
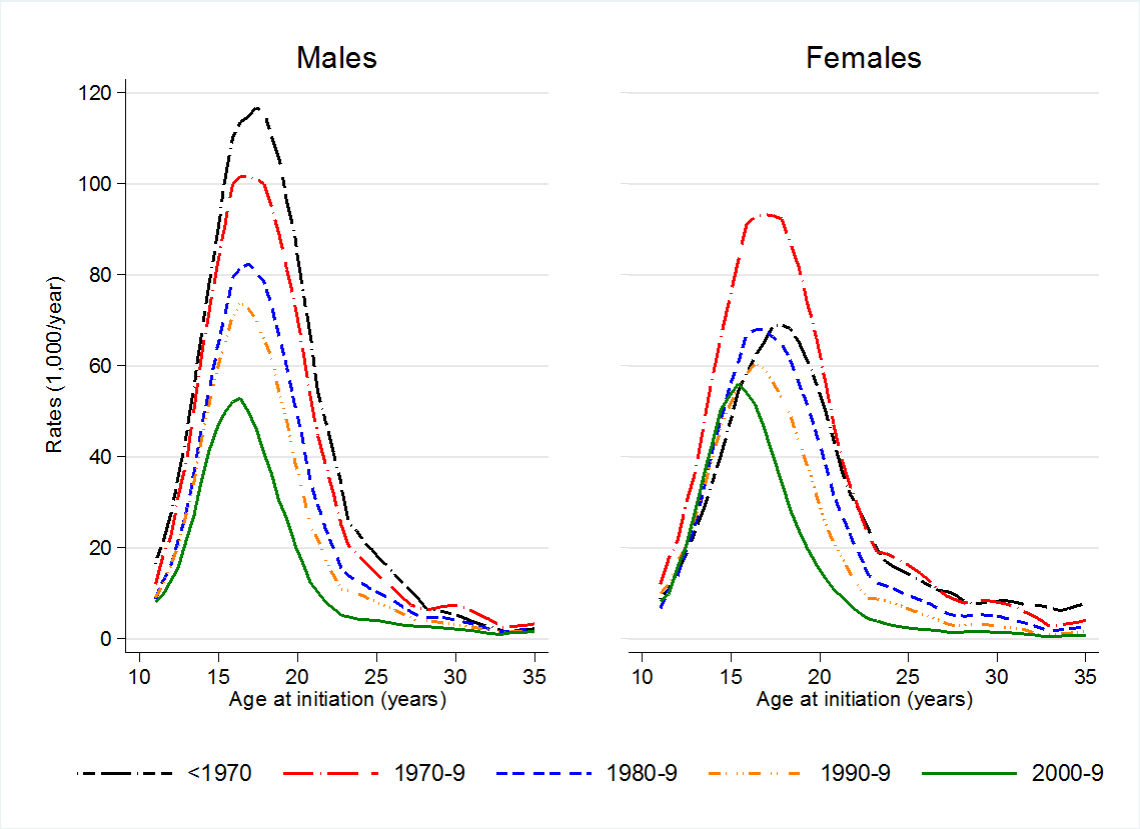
Crude rates of smoking initiation according to age, by sex and period. All European regions combined.

The crude rates of smoking initiation by region, age and time period are shown in S3 Table (males) and S4 Table (females). In all the regions combined, the rates of smoking initiation before 1970 peaked at 18 years of age in males and 19 in females; in the 2000s, they peaked at ages 16 and 15, respectively (Fig 1).

**Fig 2:**
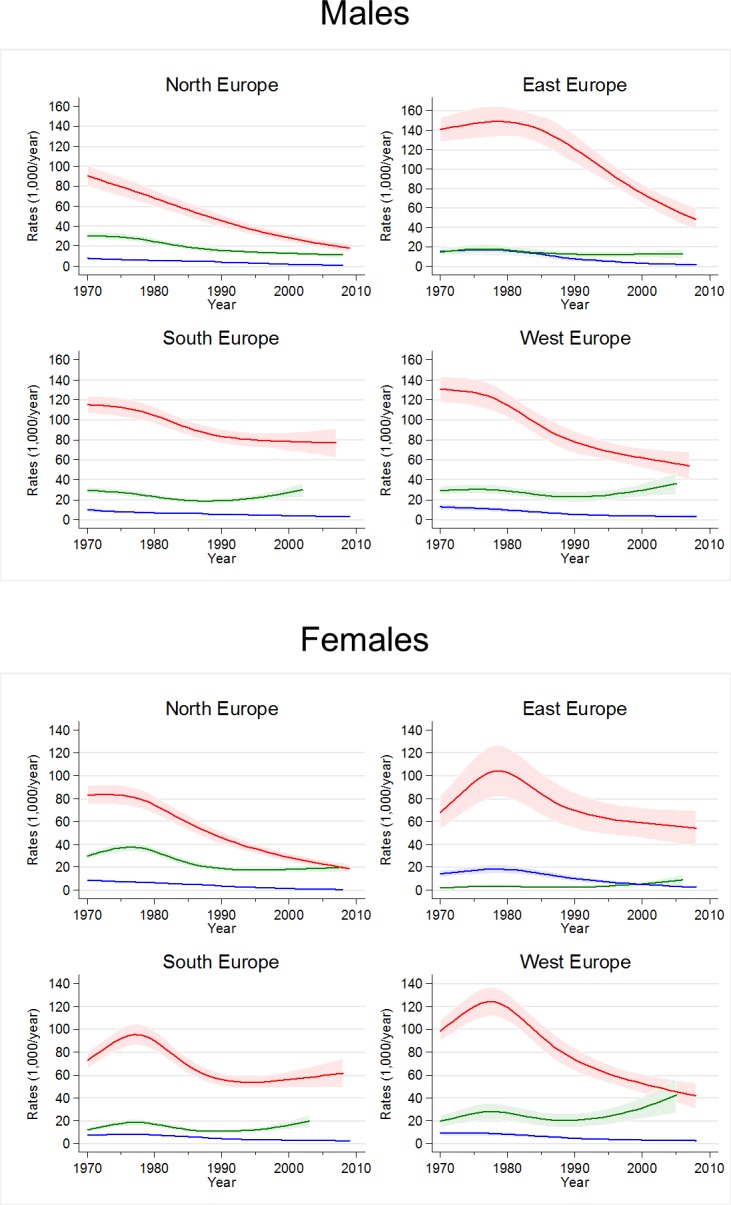
Estimated trends in smoking initiation by region with 95% confidence intervals (1970–2009). Top panel: males. Bottom panel: women. Green lines: age 11–15 years. Red lines: age 16–20 years. Blue lines: age 21–35 years. Countries represented are Denmark, Finland, Iceland, Norway, Sweden, United Kingdom (North Europe); Estonia, Macedonia, Poland (East Europe); Italy, Portugal, Spain (South Europe); Belgium, France, Germany, Netherlands, Switzerland (West Europe).

Fig 2 shows adjusted smoothed trends in smoking initiation in males (top panel) and females (bottom panel) across age groups and by region. Overall, initiation rates were highest during late adolescence (16–20 years, red lines). In this age group, in 1970, rates ranged from 90 (North Europe) to 140 (East Europe) per 1000/year in males, and from 70 (East and South Europe) to 100 (West Europe) per 1000/year in females. In late adolescent males from North Europe, rates decreased steadily throughout the study period. In the other groups, rates decreased only after they had reached a peak during the 1970s. In South Europe the decline levelled off after 1990 for both sexes. Around 2005, initiation rates during late adolescence were the lowest in males and females from North Europe (20 per 1000/year) and the highest in males from South Europe (80 per 1000/year).

Initiation rates in early adolescence (11–15 years, green lines) were lower than in late adolescence, but they started to increase after 1990 in all regions, except for males in North Europe who had stable rates. In the 2000s, initiation rates during early adolescence were above 20 per 1000/year in males from South and West Europe, as well as in females from West Europe. In the latter group, initiation rates reached 40 per 1000/year by 2005, similar to those of males of the same age and older females (16–20 years).

In young adults (21–35 years, blue lines), initiation rates were generally low (below 20 per 1000/year) and they were either stable or declining over time.

### Age-Period-Cohort analysis

The APC analysis suggested the presence of birth cohort effects (S3 Fig). In fact, smoking initiation rates for subjects born in the 1950s and subjects born after 1980 from East and West Europe (both sexes) and North Europe (females) were higher than expected under the hypothesis of additivity of age and period effects, as indicated by positive model residuals. These results are in agreement with the age-stratified analysis. For example, the excess of smoking initiation (positive residuals) in the youngest birth cohorts of West European females (S3 Fig) was mirrored by the different time trends between young adolescents (upward trend) and older subjects (downward trend) in the age-stratified analysis (Fig 2).

## Discussion

We investigated long-term trends in the uptake of regular smoking across Europe. We found that smoking initiation rates were highest during late adolescence. In this age group, rates decreased substantially between 1970 and 2009 across Europe, with the exception of South Europe where this decline levelled off after 1990. The most crucial finding of our work was the marked increase in smoking initiation during early adolescence observed after 1990, for both sexes and in all regions, except for North European males. This finding was corroborated by the APC analysis, which highlighted the presence of birth cohort effects in the youngest cohorts. Our study also suggests that if youngsters reach age 20 as non-smokers, they are very unlikely to start later in life.

Our findings of the highest initiation rates during late adolescence are in agreement with Eurobarometer surveys showing that the majority of European smokers (53% according to the 2015 report) start between the ages of 15 and 18 years.[1] The European School Survey Project on Alcohol and Other Drugs (ESPAD) documented a global decrease in the prevalence of lifetime use of cigarettes among 15–16 year-olds between 1995 (67%) and 2015 (47%).[6] In line with this trend, we found a marked reduction in smoking initiation since the 1970s. This seems to suggest that the tobacco control policies implemented over time, together with the dissemination of information on the harmful effects of smoking, have effectively addressed the age group that is at the greatest risk of starting smoking. In North Europe, initiation rates during late adolescence reached a minimum of 20 per 1000/year around 2005 in both sexes, while in the rest of Europe they ranged from 40 (West European females) to 80 (South European males) per 1000/year. Moreover, the declining trend levelled off in South Europe after 1990. These findings suggest that, in East, South and West European countries, the strategies to prevent adolescents from smoking are lagging behind North Europe. Scandinavia and the UK are at the forefront of tobacco control policies and scientific research on tobacco control.[2,23,24] Scandinavian countries were the first in Europe to adopt anti-tobacco initiatives in the 1970s.[25]

We found a worrying increase in smoking initiation during early adolescence after 1990, in spite of the reduction observed among older subjects. In line with our findings, the proportion of European smokers who start before the age of 15 years has been reported to increase from 17% to 19% between 2012 and 2014.[1] Similar trends towards an anticipation in smoking initiation have also been reported in the US.[8] Tobacco use in young adolescents is strongly influenced by siblings and friends.[26] Early smoking initiation is associated with early puberty onset, possibly because of a gap between physical and social maturity,[27] and age at puberty has been decreasing in both sexes in Europe and elsewhere.[28]

An increase in tobacco prices may be a successful strategy for young people, who are the most price-sensitive sector of the population.[29–31] Current policies on prices focus on conventional (boxed) cigarettes, and they seem to induce a shift to cheaper products, including hand-rolled cigarettes.[30] Equalising taxation levels of all tobacco products, as advocated by the Framework Convention on Tobacco Control, may limit smoking initiation especially during early adolescence.[2] Smoking in youth can be reduced by preventing tobacco sales to minors through law enforcement, which may include warnings and fines to non-compliant retailers.[32,33] Since young adolescents generally obtain cigarettes from their peers, restricting sales to minors can break the chain of tobacco supply to them [12]. Reducing exposure to cigarette advertising (including pack display at the point of sale), and implementing plain packaging in combination with pictorial health warnings, makes youth less likely to try smoking or to smoke again after experimentation.[34–36] It is still unclear whether smoking bans can reduce the prevalence of smoking, but smoke-free places may produce an environment that is less favourable to experimentation in young adolescents.[37]

### Our findings in the context of current tobacco control policies

**Fig 3:**
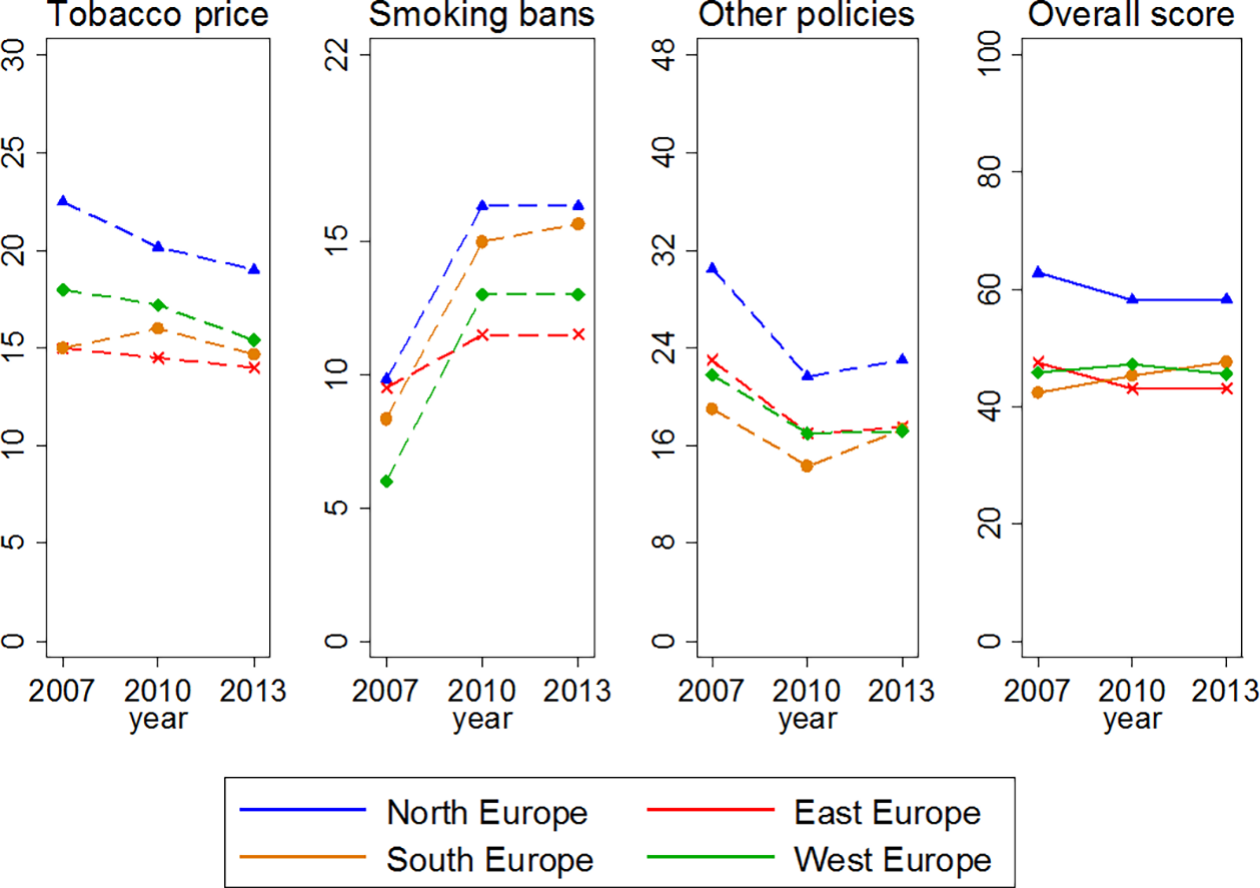
Trends in implementation of tobacco control policies by European region (2007–2013). Plots obtained using the Tobacco Control Scale scoring system from Joossens & Raw [23,38,39]. Markers indicate mean scores for the countries included in the study, with the exception of Macedonia in East Europe (not available). Y-axes show theoretical ranges. “Other policies” is the combination of spending for public information campaigns, bans on advertising, health warning labels, and treatment for smoking cessation.

Since we were able to estimate trends only until 2005–2009, we looked at available data on tobacco control policies in order to generalise our findings to present. We used the Tobacco Control Scale (TCS), a quantitative scoring system based on expert opinions that evaluates policies on tobacco prices, smoke-free places, spending on information campaigns, bans on advertising, health warning labels and treatment for smoking cessation.[23,38,39] According to the TCS, no European region showed a clear progress in policy implementation between 2007 and 2013 (Fig 3). Even if North Europe had the best performance, there is large room for improvement in all regions: on average, TCS scores for 2013 were between 40 and 60 in a scale ranging up to 100. Therefore, we speculate that major reductions in the rates of initiation are unlikely to have occurred in the same period.

### Strengths and limitations

Strengths of our study include the use of large samples of European populations, a consistent assessment of age at initiation across all the studies, and a long time window of observation. A limitation is that we used multiple random samples from studies with different designs and age ranges in a number of countries, which may not be representative of the European population. Participation rates varied widely across studies and centres, which could have introduced some bias. However, a sensitivity analysis excluding samples with participation rates below 40% produced consistent results. Our data had not been collected to specifically assess smoking trends. We applied an operational definition of smoking that focused on regular smoking but we were not able to investigate smoking experimentation. The questions available in RHINE did not address regular smoking but excluding this study, which contributed with 18.0 and 8.6% of the subjects from North and East Europe respectively, did not make a material difference in the results. The retrospective assessment of age at initiation may have introduced recall bias, although we found that reporting was fairly consistent up to two decades apart (S1 Appendix).

Since we could not assess trends in smoking intensity, it may be argued that the observed trends were driven by trends in occasional smoking. However, our findings were confirmed in a sensitivity analysis excluding subjects reporting to smoke 5 or less cigarettes/day. We had no data to distinguish between boxed cigarettes, hand-rolled cigarettes, or e-cigarettes. However, boxed cigarettes are still the most common tobacco product in Europe, and most of our data come from a period preceding the widespread availability of e-cigarettes.[1] Finally, we were not able to use multilevel models in the APC analyses due to data sparseness.

### Conclusions

Our findings suggest that the rates of smoking initiation are still unacceptably high at ages 16–20 years in most European regions, and they are increasing among those aged 15 years or younger. In West Europe, females aged 11–15 years seem to deserve particular attention, since their rates of initiation have reached the rates observed in females aged 16–20 years. Some children experiment with smoking because they feel pressured by peers, and parental smoking may give easy access to cigarettes. These social factors are difficult to address and require complex community-level interventions.[13] However, there is a necessity for countries to intensify implementation of evidence-based tobacco control measures reaching adolescents, especially price increases, enforced legislation on sales to minors, plain packaging, and bans on advertising. The use of internet and social media campaigns may also represent a valuable strategy to target the youngsters.[40]

## Supporting information

S1 Appendix Comparison of age at smoking initiation reported at different study waves in ECRHS and ISAYA. (DOCX)

S2 Appendix Supplementary information on the original studies. (DOCX)

S1 Dataset Minimal data set to replicate the analyses. (CSV)

S1 Table Distribution of the subjects by centre and study. ^a^ ECRHS, European Community Respiratory Health Survey; RHINE, Respiratory Health in Northern Europe study; GA^2^LEN, Global Allergy and Asthma Network of Excellence study; ISAYA, Italian Study on Asthma in Young Adults; GEIRD, Gene Environment Interactions in Respiratory Diseases study.
(DOCX)

S2 Table Number of samples, participation rates, and characteristics of participants by study. ^a^ original study samples, which correspond to centres (or centres crossed by age group in GEIRD); see S1 Table. ^b^ participation rates at the first wave (baseline) for studies with follow up data. In the case of ECRHS clinical, ECRHS Italy and RHINE, which were follow-up studies of ECRHS I stage 1 (S2 Fig), participation rates were obtained by multiplication assuming independence of participation between ECRHS I stage 1 and the consecutive study. ^c^ age at baseline for studies with follow up data.
(DOCX)

S3 Table Crude rates of smoking initiation per 1000/year (and person-years at risk) in males by region, age group, and period. ^a^ Countries represented are Denmark, Finland, Iceland, Norway, Sweden, United Kingdom (North Europe); Estonia, Macedonia, Poland (East Europe); Italy, Portugal, Spain (South Europe); Belgium, France, Germany, Netherlands, Switzerland (West Europe). ^b^ 1^st^ percentile in the class (combining all sexes and regions) was 1944.
(DOCX)

S4 Table Crude rates of smoking initiation per 1000/year (and person-years at risk) in females by region, age group, and period. ^a^ Countries represented are Denmark, Finland, Iceland, Norway, Sweden, United Kingdom (North Europe); Estonia, Macedonia, Poland (East Europe); Italy, Portugal, Spain (South Europe); Belgium, France, Germany, Netherlands, Switzerland (West Europe). ^b^ 1^st^ percentile in the class (combining all sexes and regions) was 1944.
(DOCX)

S1 Fig Distribution of participants by study and year. ^**a** a^ Grey boxes: subjects identified either in cross-sectional studies or at the first wave of cohort studies; hollow boxes: subjects with follow-up data.
(DOCX)

S2 Fig Flow-chart showing the different stages and waves of the ECRHS study (in italics), and the three cohorts that stem from the ECRHS study (in bold). ^a^ data not used for the analysis (no information on smoking histories was available). ^b^ data not used for the analysis (the question on age at smoking initiation was different from the questions used in the other studies).
(DOCX)

S3 Fig Cohort effects in males (blue dots) and females (red dots). ^**a** a^ APC modelling. The dots represent deviance residuals from Age-Period models according to Cohort (birth year). The fitting lines were obtained by local polynomial smoothing for a visual purpose. P-values are from Wald tests: the null hypothesis is that the regression coefficients for birth year (natural splines with three knots) are jointly zero; accordingly small p-values support the presence of cohort effects. ^b^ An outlier residual of value 4.73 at birth year 1954 was excluded for a graphical reason.
(DOCX)

## Abbreviations

APC: Age-Period-Cohort
ALEC: Ageing Lungs In European Cohorts
ECRHS: European Community Respiratory Health Survey
GA2LEN: Global Allergy and Asthma Network of Excellence
GEIRD: Gene Environment Interactions in Respiratory Diseases
ISAYA: Italian Study on Asthma in Young Adults
RHINE: Respiratory Health in Northern Europe
TCS: Tobacco Control Scale
