# Supplementary material for: Trends in smoking initiation in Europe over 40 years: A retrospective cohort study
Source: PLoS One. 2018 Aug 22;13(8):e0201881. doi: 10.1371/journal.pone.0201881 (PMC6104979; doi:10.1371/journal.pone.0201881)
Supplement: S1 Appendix — (DOCX) [file pone.0201881.s001.docx]

Marcon A, et al. Trends in smoking initiation in Europe over 40 years: a retrospective cohort study

**S1 Appendix. Comparison of age at smoking initiation reported at different study waves in ECRHS and ISAYA.**

In all the studies, age at smoking initiation was based on the question “How old were you when you started smoking?”, which was asked to regular ever smokers only. For subjects with follow-up data, we derived age at smoking initiation using the first information available *a priori*, assuming a more accurate recall of smoking initiation when a subject was younger compared to the information reported at an older age.

We compared age at initiation reported at different waves of cohort studies using the data from ECRHS (3 waves) and ISAYA (2 waves), where participants had the youngest median age at the 1^st^ wave (see S2 Table). We restricted this analysis to the subjects who had reported to be regular ever smokers at both study waves under comparison, and stratified the analysis by age at the 1^st^ study wave.

This analysis (see Table below) showed that 67–70% of smokers reported the same age at smoking initiation (±1 year) at two consecutive study waves up to two decades apart. Mean ages at initiation reported at different waves were similar. For example, subjects aged <30 years at ECRHS I reported that they had started smoking at age 16.2 years on average in ECRHS I, and at age 16.5 years in ECRHS III, which corresponds to a 0.3-year mean difference between waves carried out 20 years apart. There were fairly good correlations between age at initiation reported at different waves, as suggested by Spearman’s ρ coefficients between 0.70 and 0.80. Finally, precision was higher, i.e. SDs were smaller, when age at initiation was derived using the first information available.

| Study waves  under comparison | | | Age at initiation at 2^nd^ wave  (*vs* 1^st^ wave) | | | Smokers aged <30 years  at the 1^st^ wave | | | | Smokers aged ≥30 years  at the 1^st^ wave | | |  |
| --- | --- | --- | --- | --- | --- | --- | --- | --- | --- | --- | --- | --- | --- |
| 1^st^ wave | 2^nd^ wave | Time between  waves ^a^ | Later initiation,  n (%) | Same  (± 1 year),  n (%) | Earlier initiation,  n (%) | N | Age at initiation  reported  at 1^st^ wave ^b^ | Age at initiation  reported  at 2^nd^ wave ^b^ | Spearman’s ρ | N | Age at initiation  reported  at 1^st^ wave ^b^ | Age at initiation  reported  at 2^nd^ wave ^b^ | Spearman’s ρ |
| ECRHS I | ECRHS II | 9 (6–11) | 559 (14%) | 2857 (70%) | 666 (16%) | 1078 | 16.2 ± 2.5 | 16.4 ± 2.8 | 0.77  (p<0.001) | 3004 | 17.1 ± 3.4 | 17.0 ± 3.7 | 0.80  (p<0.001) |
| ECRHS I | ECRHS III | 20 (16–23) | 384 (16%) | 1615 (67%) | 427 (18%) | 629 | 16.2 ± 2.4 | 16.5 ± 3.2 | 0.70  (p<0.001) | 1797 | 17.1 ± 3.3 | 17.0 ± 3.9 | 0.76  (p<0.001) |
| ISAYA I | ISAYA II | 10 (8–11) | 150 (16%) | 634 (67%) | 161 (17%) | 308 | 16.8 ± 2.5 | 17.0 ± 3.1 | 0.70  (p<0.001) | 637 | 17.1 ± 3.5 | 17.0 ± 3.5 | 0.74  (p<0.001) |

^a^ median (min–max)

^b^ mean ± standard deviation
