# Supplementary material for: Trends in smoking initiation in Europe over 40 years: A retrospective cohort study
Source: PLoS One. 2018 Aug 22;13(8):e0201881. doi: 10.1371/journal.pone.0201881 (PMC6104979; doi:10.1371/journal.pone.0201881)
Supplement: S1 Table — a ECRHS, European Community Respiratory Health Survey; RHINE, Respiratory Health in Northern Europe study; GA2LEN, Global Allergy and Asthma Network of Excellence study; ISAYA, Italian Study on Asthma in Young Adults; GEIRD, Gene Environment Interactions in Respiratory Diseases study. (DOCX) [file pone.0201881.s004.docx]

Marcon A, et al. Trends in smoking initiation in Europe over 40 years: a retrospective cohort study

**S1 Table. Distribution of the subjects by centre and study.**

| **Region** | **Country** | **Centre** | **Study ^a^** |  |  |  |  |  | | |
| --- | --- | --- | --- | --- | --- | --- | --- | --- | --- | --- |
|  |  |  | **ECRHS** | **ECRHS Italy** | **RHINE** | **GA^2^LEN** | **ISAYA** | **GEIRD** | | |
|  |  |  |  |  |  |  |  | **20–44 y** | **45–64 y** | **65–84 y** |
| **North Europe** | Denmark | Aarhus | 392 |  | 1943 |  |  |  |  |  |
|  |  | Odense |  |  |  | 3336 |  |  |  |  |
|  | Finland | Helsinki |  |  |  | 1824 |  |  |  |  |
|  | Iceland | Reykjavik | 562 |  | 1445 |  |  |  |  |  |
|  | Norway | Bergen | 834 |  | 1529 |  |  |  |  |  |
|  |  | Oslo |  |  |  | 1527 |  |  |  |  |
|  | Sweden | Gothenburg | 680 |  | 1202 | 8440 |  |  |  |  |
|  |  | Stockholm |  |  |  | 5853 |  |  |  |  |
|  |  | Umea | 552 |  | 1523 | 6106 |  |  |  |  |
|  |  | Uppsala | 621 |  | 1449 | 6153 |  |  |  |  |
|  | UK | Caerphilly | 376 |  |  |  |  |  |  |  |
|  |  | Ipswich | 448 |  |  |  |  |  |  |  |
|  |  | London |  |  |  | 1971 |  |  |  |  |
|  |  | Norwich | 473 |  |  |  |  |  |  |  |
|  |  | Southampton |  |  |  | 1337 |  |  |  |  |
| **East Europe** | Estonia | Tartu | 411 |  | 903 |  |  |  |  |  |
|  | Macedonia | Skopje |  |  |  | 3614 |  |  |  |  |
|  | Poland | Katowice |  |  |  | 2633 |  |  |  |  |
|  |  | Krakow |  |  |  | 1256 |  |  |  |  |
|  |  | Lodz |  |  |  | 1739 |  |  |  |  |

Marcon A, et al. Trends in smoking initiation in Europe over 40 years: a retrospective cohort study

| **Region** | **Country** | **Centre** | **Study ^a^** |  |  |  |  |  |  |  |
| --- | --- | --- | --- | --- | --- | --- | --- | --- | --- | --- |
|  |  |  | **ECRHS** | **ECRHS Italy** | **RHINE** | **GA^2^LEN** | **ISAYA** | **GEIRD** |  |  |
|  |  |  |  |  |  |  |  | **20–44 y** | **45–64 y** | **65–84 y** |
| **South Europe** | Italy | Ancona |  |  |  |  |  | 1834 |  |  |
|  |  | Ferrara |  |  |  |  | 2100 |  |  |  |
|  |  | Palermo |  |  |  | 968 |  |  |  |  |
|  |  | Pavia | 309 | 701 |  |  | 2444 | 945 | 447 |  |
|  |  | Pisa |  |  |  |  | 2404 |  |  |  |
|  |  | Salerno |  |  |  |  |  | 1744 |  |  |
|  |  | Sassari |  |  |  |  | 2048 | 1232 | 516 | 423 |
|  |  | Sassuolo |  |  |  |  | 2118 |  |  |  |
|  |  | Siracusa |  |  |  |  | 1177 |  |  |  |
|  |  | Terni |  |  |  |  |  | 1622 |  |  |
|  |  | Torino | 244 | 1255 |  |  | 2236 | 1169 | 483 |  |
|  |  | Udine |  |  |  |  | 2071 |  |  |  |
|  |  | Verona | 342 | 1728 |  |  | 2150 | 1722 | 660 | 566 |
|  | Portugal | Coimbra |  |  |  | 2199 |  |  |  |  |
|  | Spain | Albacete | 435 |  |  |  |  |  |  |  |
|  |  | Barcelona | 390 |  |  |  |  |  |  |  |
|  |  | Galdakao | 486 |  |  |  |  |  |  |  |
|  |  | Huelva | 270 |  |  |  |  |  |  |  |
|  |  | Oviedo | 356 |  |  |  |  |  |  |  |
| **West Europe** | Belgium | Antwerp City | 564 |  |  |  |  |  |  |  |
|  |  | Antwerp South | 556 |  |  |  |  |  |  |  |
|  |  | Ghent |  |  |  | 1855 |  |  |  |  |
|  | France | Grenoble | 473 |  |  |  |  |  |  |  |
|  |  | Montpellier |  |  |  | 1300 |  |  |  |  |
|  | Germany | Brandenburg |  |  |  | 2237 |  |  |  |  |
|  |  | Erfurt | 731 |  |  |  |  |  |  |  |
|  |  | Hamburg | 1252 |  |  |  |  |  |  |  |
|  |  | Munich |  |  |  | 2015 |  |  |  |  |
|  | Netherlands | Amsterdam |  |  |  | 3129 |  |  |  |  |
|  |  | Bergen op Zoom | 451 |  |  |  |  |  |  |  |
|  |  | Geleen | 395 |  |  |  |  |  |  |  |
|  |  | Groningen | 378 |  |  |  |  |  |  |  |
|  | Switzerland | Basel | 842 |  |  |  |  |  |  |  |

^a^ ECRHS, European Community Respiratory Health Survey; RHINE, Respiratory Health in Northern Europe study; GA^2^LEN, Global Allergy and Asthma Network of Excellence study; ISAYA, Italian Study on Asthma in Young Adults; GEIRD, Gene Environment Interactions in Respiratory Diseases study.
