# Supplementary material for: Trends in smoking initiation in Europe over 40 years: A retrospective cohort study
Source: PLoS One. 2018 Aug 22;13(8):e0201881. doi: 10.1371/journal.pone.0201881 (PMC6104979; doi:10.1371/journal.pone.0201881)
Supplement: S2 Table — a original study samples, which correspond to centres (or centres crossed by age group in GEIRD); see S1 Table. b participation rates at the first wave (baseline) for studies with follow up data. In the case of ECRHS clinical, ECRHS Italy and RHINE, which were follow-up studies of ECRHS I stage 1 (S2 Fig), participation rates were obtained by multiplication assuming independence of participation between ECRHS I stage 1 and the consecutive study. c age at baseline for studies with follow up data. (DOCX) [file pone.0201881.s005.docx]

Marcon A, et al. Trends in smoking initiation in Europe over 40 years: a retrospective cohort study

**S2 Table**. **Number of samples, participation rates, and characteristics of participants by study.**

| Study | ECRHS clinical | ECRHS Italy | RHINE | GA^2^LEN | ISAYA | GEIRD |
| --- | --- | --- | --- | --- | --- | --- |
| Samples^a^ (n) | 27 | 3 | 7 | 20 | 9 | 13 |
| Participation rate^b^, %  (median, min–max) | 54.4  (30.2–83.1) | 58.1  (48.5–73.9) | 48.2  (38.2–53.5) | 42.8  (25.4–80.4) | 74.0  (48.7–82.2) | 59.1  (37.1–67.7) |
| Subjects (n) | 13,823 | 3,684 | 9,994 | 59,492 | 18,748 | 13,363 |
| Males (%) | 48.0 | 48.6 | 46.9 | 44.8 | 49.3 | 48.4 |
| Birth cohort, year  (median, min–max) | 1958  (1945–73) | 1958  (1946–71) | 1959  (1945–73) | 1965  (1908–94) | 1966  (1953–80) | 1969  (1925–89) |
| Age^c^, year  (median, min–max) | 33  (20–47) | 41  (28–60) | 52  (37–67) | 43  (14–100) | 33  (19–47) | 39  (20–86) |
| Region (%)  North Europe  East Europe  South Europe  West Europe | 35.7  3.0  20.5  40.8 | -  -  100  - | 91.0  9.0  -  - | 61.4  15.5  5.3  17.7 | -  -  100  - | -  -  100  - |
| Ever smokers (%) | 61.3 | 52.0 | 52.6 | 42.8 | 48.6 | 46.2 |
| Age at starting smoking, year  (mean±SD) | 17.0±3.6 | 17.6±3.6 | 17.3±4.4 | 17.3±4.4 | 17.2±3.2 | 17.6±4.0 |
| Total years at risk (in  age range 11–35 years) | 187,020 | 57,756 | 157,606 | 876,740 | 268,755 | 207,800 |

^a^ original study samples, which correspond to centres (or centres crossed by age group in GEIRD); see S1 Table

^b^ participation rates at the first wave (baseline) for studies with follow up data. In the case of ECRHS clinical, ECRHS Italy and RHINE, which were follow-up studies of ECRHS I stage 1 (S2 Fig), participation rates were obtained by multiplication assuming independence of participation between ECRHS I stage 1 and the consecutive study

^c^ age at baseline for studies with follow up data
