# Supplementary material for: Trends in smoking initiation in Europe over 40 years: A retrospective cohort study
Source: PLoS One. 2018 Aug 22;13(8):e0201881. doi: 10.1371/journal.pone.0201881 (PMC6104979; doi:10.1371/journal.pone.0201881)
Supplement: S3 Table — a Countries represented are Denmark, Finland, Iceland, Norway, Sweden, United Kingdom (North Europe); Estonia, Macedonia, Poland (East Europe); Italy, Portugal, Spain (South Europe); Belgium, France, Germany, Netherlands, Switzerland (West Europe). b 1st percentile in the class (combining all sexes and regions) was 1944. (DOCX) [file pone.0201881.s006.docx]

Marcon A, et al. Trends in smoking initiation in Europe over 40 years: a retrospective cohort study

**S3 Table. Crude rates of smoking initiation per 1000/year (and person-years at risk) in males by region, age group, and period.**

|  |  | Period | | | | |
| --- | --- | --- | --- | --- | --- | --- |
| Region ^a^ | Age  (years) | <1970 ^b^ | 1970–9 | 1980–9 | 1990–9 | 2000–9 |
| North  Europe | 11–15 | 45.1  (43413) | 39.8  (26377) | 29.8  (18690) | 24.8  (13583) | 15.2  (9256) |
|  | 16–20 | 111.1  (19791) | 73.5  (17493) | 52.1  (19151) | 35.0  (12105) | 18.1  (10035) |
|  | 21–25 | 25.1  (7595) | 14.6  (11816) | 12.5  (16223) | 7.1  (13040) | 3.2  (9707) |
|  | 26–30 | 5.2  (3686) | 5.1  (8584) | 4.3  (13156) | 3.4  (15525) | 2.1  (9614) |
|  | 31–35 | 1.3  (1543) | 1.9  (5323) | 1.3  (10996) | 1.3  (15026) | 0.9  (11348) |
| East  Europe | 11–15 | 16.6  (7989) | 32.9  (4498) | 26.2  (4127) | 24.7  (4005) | 29.2  (2359) |
|  | 16–20 | 129.5  (4100) | 144.2  (3245) | 134.9  (3040) | 97.0  (2774) | 64.4  (2515) |
|  | 21–25 | 56.8  (1409) | 53.9  (1632) | 40.5  (1681) | 18.5  (1896) | 8.9  (1912) |
|  | 26–30 | 2.6  (763) | 15.7  (1017) | 10.6  (1413) | 3.6  (1660) | 3.1  (1634) |
|  | 31–35 | 0.0  (339) | 7.9  (762) | 2.3  (1282) | 1.4  (1454) | 1.4  (1474) |
| South  Europe | 11–15 | 41.3  (19455) | 38.7  (32872) | 31.1  (33343) | 37.0  (10341) | 60.5  (1024) |
|  | 16–20 | 112.2  (6579) | 117.0  (15335) | 89.9  (26792) | 80.8  (15356) | 63.3  (3082) |
|  | 21–25 | 26.7  (2514) | 24.3  (5260) | 13.4  (15946) | 12.7  (18287) | 8.1  (4336) |
|  | 26–30 | 7.4  (1488) | 7.5  (2260) | 5.0  (9438) | 5.2  (18543) | 4.2  (5465) |
|  | 31–35 | 1.4  (727) | 0.0  (1496) | 1.7  (4758) | 1.6  (14333) | 1.9  (7011) |
| West  Europe | 11–15 | 35.2  (13938) | 42.0  (9306) | 39.2  (4920) | 30.9  (4953) | 66.4  (2350) |
|  | 16–20 | 148.8  (6163) | 138.5  (5330) | 94.8  (5477) | 82.6  (1720) | 67.1  (3905) |
|  | 21–25 | 29.8  (1911) | 33.2  (3130) | 19.9  (4228) | 12.5  (2409) | 6.5  (1996) |
|  | 26–30 | 16.1  (933) | 7.3  (2184) | 6.2  (3223) | 3.8  (3194) | 6.3  (1109) |
|  | 31–35 | 3.3  (302) | 7.6  (1313) | 2.6  (2739) | 1.5  (3270) | 1.7  (1743) |

^a^ Countries represented are Denmark, Finland, Iceland, Norway, Sweden, United Kingdom (North Europe); Estonia, Macedonia, Poland (East Europe); Italy, Portugal, Spain (South Europe); Belgium, France, Germany, Netherlands, Switzerland (West Europe).

^b^ 1^st^ percentile in the class (combining all sexes and regions) was 1944
