# Supplementary material for: Trends in smoking initiation in Europe over 40 years: A retrospective cohort study
Source: PLoS One. 2018 Aug 22;13(8):e0201881. doi: 10.1371/journal.pone.0201881 (PMC6104979; doi:10.1371/journal.pone.0201881)
Supplement: S4 Table — a Countries represented are Denmark, Finland, Iceland, Norway, Sweden, United Kingdom (North Europe); Estonia, Macedonia, Poland (East Europe); Italy, Portugal, Spain (South Europe); Belgium, France, Germany, Netherlands, Switzerland (West Europe). b 1st percentile in the class (combining all sexes and regions) was 1944. (DOCX) [file pone.0201881.s007.docx]

Marcon A, et al. Trends in smoking initiation in Europe over 40 years: a retrospective cohort study

**S4 Table. Crude rates of smoking initiation per 1000/year (and person-years at risk) in females by region, age group, and period.**

|  |  | Period | | | | |
| --- | --- | --- | --- | --- | --- | --- |
| Region ^a^ | Age  (years) | <1970 ^b^ | 1970–9 | 1980–9 | 1990–9 | 2000–9 |
| North  Europe | 11–15 | 28.8  (48867) | 51.0  (30088) | 37.0  (23024) | 29.0  (19057) | 25.5  (11044) |
|  | 16–20 | 85.0  (26078) | 84.5  (19051) | 57.1  (21564) | 38.6  (14930) | 20.1  (13244) |
|  | 21–25 | 22.0  (11356) | 16.3  (14461) | 10.9  (17056) | 6.1  (15149) | 2.8  (13112) |
|  | 26–30 | 7.8  (5933) | 6.8  (11367) | 4.3  (14256) | 2.1  (17345) | 0.3  (11822) |
|  | 31–35 | 4.9  (2663) | 2.6  (7371) | 1.9  (13385) | 1.1  (15936) | 0.4  (13335) |
| East  Europe | 11–15 | 2.9  (10332) | 7.3  (5768) | 7.5  (5208) | 11.2  (5179) | 22.3  (2467) |
|  | 16–20 | 47.7  (6564) | 91.7  (5376) | 84.9  (4583) | 72.3  (4094) | 67.3  (3252) |
|  | 21–25 | 22.5  (3289) | 36.4  (3678) | 31.5  (3115) | 20.3  (3347) | 10.7  (2804) |
|  | 26–30 | 9.5  (1996) | 18.1  (2485) | 13.0  (3088) | 5.0  (2978) | 2.4  (2495) |
|  | 31–35 | 6.8  (740) | 5.9  (2044) | 4.3  (2991) | 3.0  (2678) | 1.1  (2643) |
| South  Europe | 11–15 | 10.4  (20793) | 28.5  (36387) | 23.6  (35523) | 24.3  (12331) | 40.1  (1370) |
|  | 16–20 | 46.8  (8849) | 95.8  (19826) | 66.0  (31075) | 55.2  (19059) | 42.5  (4256) |
|  | 21–25 | 13.4  (3744) | 17.7  (9224) | 11.3  (20403) | 9.9  (23318) | 5.3  (6363) |
|  | 26–30 | 4.8  (2080) | 8.8  (4644) | 5.1  (13242) | 3.9  (23647) | 2.3  (8397) |
|  | 31–35 | 3.5  (1141) | 1.8  (2244) | 1.3  (8391) | 1.1  (18514) | 1.2  (10205) |
| West  Europe | 11–15 | 15.5  (16143) | 37.9  (10444) | 32.6  (6006) | 39.9  (6895) | 80.2  (3019) |
|  | 16–20 | 76.8  (8988) | 119.4  (7037) | 87.5  (6410) | 76.0  (2486) | 48.7  (5299) |
|  | 21–25 | 16.5  (3811) | 25.8  (5154) | 18.5  (4931) | 9.8  (3267) | 4.4  (2926) |
|  | 26–30 | 7.9  (2035) | 8.0  (4000) | 5.8  (4471) | 3.5  (4052) | 6.4  (1711) |
|  | 31–35 | 5.2  (772) | 1.9  (2651) | 1.9  (4650) | 1.3  (3987) | 1.2  (2528) |

^a^ Countries represented are Denmark, Finland, Iceland, Norway, Sweden, United Kingdom (North Europe); Estonia, Macedonia, Poland (East Europe); Italy, Portugal, Spain (South Europe); Belgium, France, Germany, Netherlands, Switzerland (West Europe).

^b^ 1^st^ percentile in the class (combining all sexes and regions) was 1944
