# Supplementary material for: Trends in smoking initiation in Europe over 40 years: A retrospective cohort study
Source: PLoS One. 2018 Aug 22;13(8):e0201881. doi: 10.1371/journal.pone.0201881 (PMC6104979; doi:10.1371/journal.pone.0201881)
Supplement: S1 Fig — a a Grey boxes: subjects identified either in cross-sectional studies or at the first wave of cohort studies; hollow boxes: subjects with follow-up data. (DOCX) [file pone.0201881.s008.docx]

Marcon A, et al. Trends in smoking initiation in Europe over 40 years: a retrospective cohort study

**S1 Fig. Distribution of participants by study and year ^a^**

^a^ Grey boxes: subjects identified either in cross-sectional studies or at the first wave of cohort studies; hollow boxes: subjects with follow-up data
