# Supplementary material for: Trends in smoking initiation in Europe over 40 years: A retrospective cohort study
Source: PLoS One. 2018 Aug 22;13(8):e0201881. doi: 10.1371/journal.pone.0201881 (PMC6104979; doi:10.1371/journal.pone.0201881)
Supplement: S2 Fig — a data not used for the analysis (no information on smoking histories was available). b data not used for the analysis (the question on age at smoking initiation was different from the questions used in the other studies). (DOCX) [file pone.0201881.s009.docx]

Marcon A, et al. Trends in smoking initiation in Europe over 40 years: a retrospective cohort study

**S2 Fig. Flow-chart showing the different stages and waves of the ECRHS study (in italics), and the three cohorts that stem from the ECRHS study (in bold).**

^a^ data not used for the analysis (no information on smoking histories was available)

^b^ data not used for the analysis (the question on age at smoking initiation was different from the questions used in the other studies)
