# Supplementary material for: Trends in smoking initiation in Europe over 40 years: A retrospective cohort study
Source: PLoS One. 2018 Aug 22;13(8):e0201881. doi: 10.1371/journal.pone.0201881 (PMC6104979; doi:10.1371/journal.pone.0201881)
Supplement: S3 Fig — a a APC modelling. The dots represent deviance residuals from Age-Period models according to Cohort (birth year). The fitting lines were obtained by local polynomial smoothing for a visual purpose. P-values are from Wald tests: the null hypothesis is that the regression coefficients for birth year (natural splines with three knots) are jointly zero; accordingly small p-values support the presence of cohort effects. b An outlier residual of value 4.73 at birth year 1954 was excluded for a graphical reason. (DOCX) [file pone.0201881.s010.docx]

Marcon A, et al. Trends in smoking initiation in Europe over 40 years: a retrospective cohort study

**S3 Fig**. **Cohort effects in males (blue dots) and females (red dots). ^a^**

^a^ APC modelling. The dots represent deviance residuals from Age-Period models according to Cohort (birth year). The fitting lines were obtained by local polynomial smoothing for a visual purpose. P-values are from Wald tests: the null hypothesis is that the regression coefficients for birth year (natural splines with three knots) are jointly zero; accordingly small p-values support the presence of cohort effects. ^b^ An outlier residual of value 4.73 at birth year 1954 was excluded for a graphical reason
